# How Life Is Lost

**Published:** 1878-03

**Authors:** 


**HOW LIFE IS LOST.**—A man died the other day at the Bellevue Hospital, after being sick over two years. On open ing the chest, there was scarcely a single inch of sound lungs on one side; the organ had broken down in one mass of corruption, and the yellow matter of consumption was dipped out with a skull, the most convenient cup at hand. He had been working in the garden one summer's day, and feeling a little tired at noon, went round to the shady side of the house and sat down to rest. A little wind was blowing, which was so very grateful to him that he indulged himself in it for some minutes, when he was taken with a chill, and never knew a well moment afterwards.

Only two days ago, one of the sweetest possible pair of black eyes came to inquire, with all the shrinking and diffidence inseparable from the occasion, what we thought of the case of a young gentleman who had applied for advice within the week, stating as a reason, that they were engaged to be married.

The young man in question had arisen one morning in early May, and dressed in very light clothing, but he was so much mistaken in the temperature of the weather, that he was soon chilled, without the means of changing his condition for some time, that is, he felt chilly for several consecutive hours, and had been an invalid ever since. The disease had made such fearful progress, that two thirds of his lungs were useless to him; and emaciation, night-sweats, harassing cough and swollen feet, made it useless to afford the encouragement of even prescribing for the case.

These two cases involve the same principle—getting chilled; one after exercise, the other by remaining cold for hours. Surely it is not hard to remember the lesson. Let every parent impress it on the mind of each child on the instant, and it may prevent the great calamity of dying childless, than which there are not many harder for the heart to bear; indeed it often fails to bear them, and breaks under the burden.

